# Comparison of arthroplasty vs. osteosynthesis for displaced femoral neck fractures: a meta-analysis

**DOI:** 10.1186/s13018-017-0629-5

**Published:** 2017-09-15

**Authors:** Feng-Jen Tseng, Wei-Tso Chia, Ru-Yu Pan, Leou-Chyr Lin, Hsian-Chung Shen, Chih-Hung Wang, Jia-Fwu Shyu, Ching-Feng Weng

**Affiliations:** 1 Department of Life Science and the Institute of Biotechnology, National Dong Hwa University, Hualien, 974 Taiwan Republic of China; 2 Department of Orthopedics, Hualien Armed Force General Hospital, Hualien, 971 Taiwan Republic of China; 3 Department of Health, Hsin Chu General Hospital, Hsinchu, 300 Taiwan Republic of China; 4 Department of Orthopaedics, Tri-Service General Hospital, National Defense Medical Center, Neihu 114, Taipei, Taiwan Republic of China; 5 Graduate Institute of Medical Science, National Defense Medical Center, Neihu 114, Taipei, Taiwan Republic of China; 6 Department of Biology and Anatomy, National Defense Medical Center, Neihu 114, Taipei, Taiwan Republic of China

**Keywords:** Meta-analysis, Displaced femoral neck fractures, Arthroplasty, Osteosynthesis, Elderly, Open reduction internal fixation

## Abstract

**Background:**

This meta-analysis compared clinical outcomes of arthroplasty vs. osteosynthesis for displaced femoral neck fractures.

**Methods:**

Meta-analysis was performed on the difference in revision rate and overall mortality between participants undergoing osteosynthesis vs. total hip arthroplasty (THA), osteosynthesis vs. hemiarthroplasty (HA), or THA vs. HA.

**Results:**

Pooled direct and indirect results indicated no significant difference in mortality between THA and HA (pooled OR = 0.87, 95% CI 0.55 to 1.38; *P* = 0.556), between THA and osteosynthesis (pooled OR = 1.17, 95% CI 0.69 to 1.99; *P* = 0.553), and between HA and osteosynthesis (pooled OR = 1.21, 95% CI 0.84 to 1.74; *P* = 0.304). Pooled direct and indirect results indicated no significant difference in revision rates between THA and HA (pooled OR = 0.90, 95% CI 0.26 to 3.19; *P* = 0.874). But, fewer revisions (OR = 0.19, 95% CI 0.10 to 0.34; *P* = 0.000) were seen in patients treated with THA than osteosynthesis and also in those treated with HA than osteosynthesis (OR = 0.12, 95% CI 0.07 to 0.20; *P* = 0.000). After excluding studies without showing normal cognition in inclusion criteria, pooled direct and indirect results also indicated no significant difference in mortality between THA, HA, and osteosynthesis. Similarly, there was no significant difference in revision rates between THA and HA, but HA and THA had significantly lower revision rates compared with osteosynthesis.

**Conclusions:**

There was no significant difference in overall mortality among osteosynthesis, HA, and THA. However, HA and THA had significantly lower revision rates compared with osteosynthesis. Results of the present study provide support for the use of hip arthroplasty to treat displaced fractures of the femoral neck.

## Background

Approximately 250,000 proximal femoral fractures occur in the USA each year, and 90% of these fractures occur in patients older than 50 years of age [1, 2]. Fractures of the femoral neck can be categorized into either non-displaced or displaced fractures in order to facilitate appropriate management, particularly in the elderly [3, 4]. Displaced femoral neck fractures are defined as unstable fractures that can impair blood supply to the femoral head, resulting in avascular necrosis [5, 6]. These fractures are associated with substantial fracture-related mortality and morbidity [6, 7]. An additional contributor to femoral head osteonecrosis involves the quality of the reduction or fracture fixation [8].

Osteosynthesis refers to the percutaneous placement of several parallel cannulated lag screws, and in the younger patient, such internal fixation is the standard treatment for displaced fractures [8]. Hip arthroplasty, on the other hand, refers to replacement of all or part of the hip joint with a prosthetic implant [6, 9] and can be divided into either total hip arthroplasty (THA) or hemiarthroplasty (HA). THA involves replacement of both the femoral head and the acetabular articular surface, whereas in HA, only the femoral head is replaced with an artificial implant, while the patient’s own acetabulum is retained [5, 6, 9].

Although internal fixation is recommended for most non-displaced fractures of the femoral neck, the optimal treatment for displaced fractures of the femoral neck is still controversial [10–12]. HA was once considered the procedure of choice for elderly patients with displaced (Garden stage III or IV) femoral neck fractures [13], but a Swedish study concluded that THA should be performed for displaced femoral neck fractures in older patients with normal mental function and high function [14], a conclusion that has been echoed in several more recent publications [15, 16]. Davison et al., on the other hand, recommended either reduction with internal fixation or cemented HA as alternative treatments for a displaced intracapsular fracture in a mobile and mentally competent patient under 80 years of age [10].

The literature also contains conflicting evidence regarding rates of mortality, major postoperative complications, and function in elderly patients with displaced femoral neck fractures treated either by internal fixation or arthroplasty [17]. In fact, the choice of surgical treatment for a displaced intracapsular fracture of the proximal femur in the elderly remains as controversial now as it was over 50 years ago when it was designated as “the unsolved fracture” [10, 12, 18]. This meta-analysis was designed, therefore, to address this controversy by comparing outcomes after internal fixation, hemiarthroplasty, and THA, with particular reference to mortality and revision rates because until now, few studies have compared these three alternative treatments [10]. In addition, due to the limited number of studies with head-to-head comparison of HA and total hip replacement, a statistical analysis (comprised of both direct and indirect comparisons) was utilized to achieve this study’s objective.

## Methods

### Selection criteria

Only English language publications of randomized controlled trials (RCTs) or prospective comparative studies of patients with displaced femoral neck fractures were included. Patient subjects had to be elderly (60 years of age or older) and capable of walking independently (without relying on another person), with or without aids prior to the injury. In addition, only studies that involved one or more comparisons of at least two types of intervention: (1) osteosynthesis vs. THA, (2) osteosynthesis vs. HA, or (3) THA vs. HA were included in the analysis.

Retrospective studies, letters, comments, editorials, case reports, technical reports, and non-English publications were excluded. In addition, any retrospective comparative study or single-arm study was excluded. Any study design that contained no numerical information about the outcomes of interest was also excluded.

### Search strategy

This meta-analysis was conducted in accordance with the PRISMA guidelines [19]. Medline, Cochrane, and Embase databases were searched until August 31, 2014. In addition, the reference lists of relevant studies were manually searched. Keywords used for the search included femoral neck, fracture, total hip replacement, internal fixation, open reduction internal fixation, ORIF, osteosynthesis, HA, and arthroplasty.

### Study selection and data extraction

Studies were identified by two independent reviewers using the designated search strategy. Where there was uncertainty regarding eligibility, a third reviewer was consulted. Data extraction was also performed by two independent reviewers, and a third reviewer was consulted for any uncertainties. The following data were extracted from studies that met the inclusion criteria: the name of the first author; year of publication; study design; number of participants in each treatment group; demographic data of participants, such as age and gender; diagnostic criteria; treatment methods; and duration of follow-up.

### Quality assessment

The Cochrane Risk of Bias Tool was utilized to assess the included studies [20]. The quality assessment was performed by the independent reviewers, and a third reviewer was consulted for any uncertainties.

### Outcome measures

The primary endpoint in the meta-analysis was the overall mortality, and the secondary endpoint was the revision rate. Economic outcomes, quality of life (QoL), and functional outcomes were not assessed. However, additional analysis was performed on a subgroup of studies involving only elderly subjects with no significant or severe cognitive impairment. This approach was used to determine if any differences existed regarding mortality and revision rates within this subgroup, who underwent either HA, THA, or osteosynthesis.

### Statistical analysis

The odds ratio (OR) and 95% confidence interval (CI) were calculated for binary outcomes and then compared between two different interventions. A chi-square-based test of homogeneity was performed, and the inconsistency index (*I* ^2^) statistic was determined. When heterogeneity existed between studies (*I* ^2^ > 50%), a random-effects model was used. Otherwise, fixed-effects models were applied. Pooled summary statistics for ORs of the individual studies were reported. Sensitivity analysis was performed by using the leave-one-out cross-validation approach [21]. In addition, publication bias was not assessed in groups of fewer than five studies because more than ten studies are required to detect funnel plot asymmetry [22]. Direct pairwise meta-analyses were performed using Comprehensive Meta-Analysis, version 2 (Biostat, Englewood, NJ). Adjusted indirect comparisons of pooled estimates using inverse variance weighting were then performed according to the methods of Bucher and colleagues using the indirect treatment comparison computer program, version 1.0 [23]. We calculated an indirect result between THA and HA groups.

## Results

### Literature search

**Fig. 1:**
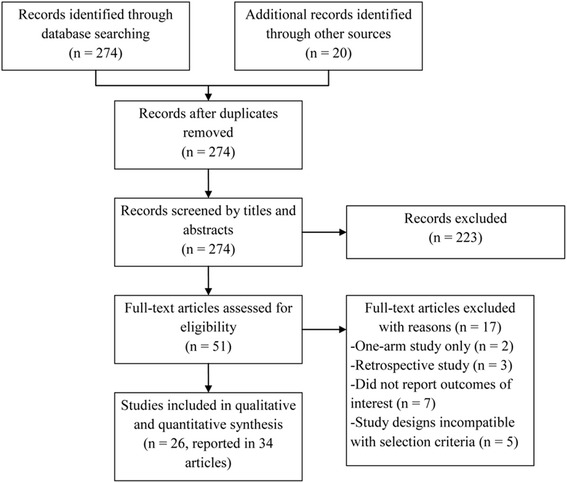
Flow chart for the study selection

After initially identifying 274 articles, 223 articles were excluded and 51 studies were assessed for eligibility. After full-text review, 17 additional articles were excluded, as shown in Fig. 1. The remaining 34 articles [10, 15, 24–55] were included in the qualitative and quantitative analyses.

### Study characteristics

Twenty-six studies (reported in the 34 articles) were included in the systematic review. All studies compared arthroplasty with osteosynthesis (Table 1); five studies (encompassing references [15, 24–29]) compared THA (*n* = 218) with osteosynthesis (*n* = 235), 14 studies (encompassing references [10, 30–44]) compared HA (*n* = 1518) with osteosynthesis (*n* = 1178), and only seven studies (encompassing references [45–55]) compared THA (*n* = 432) and HA (*n* = 462). Postoperative follow-up ranged from 14.5 months [26] to 17 years [15].

**Table 1:** A list of included studies and demography of the study subjects

First author	Normal cognition	Interventions	No. of patients	Age (years)	Male (%)	Duration of follow-up	Tools for functional measurement
Total hip arthroplasty vs. hemiarthroplasty							
Avery (2011) [45] and Baker (2006) [46]	Yes	Total hip arthroplasty	40	74	20	8.83 (7.2 to 10.3) years	Oxford Hip Score
		Hemiarthroplasty	41	76	22	8.6 (7.2 to 10) years	
Cadossi (2013) [49]	Yes	Total hip arthroplasty	42	82	19	28.6 (22 to 52) months	Harris Hip Score
		Hemiarthroplasty	41	84	32	30.1 (23 to 50) months	
Hedbeck (2011) [48] and Blomfeldt (2007) [47]	Yes	Total hip arthroplasty	60	81	22	4 years	Harris Hip Score
		Hemiarthroplasty	60	81	10		
Keating (2006) [51] and Keating (2005) [50]	Yes	Total hip arthroplasty	69	75	25	2 years	Hip rating questionnaire
		Hemiarthroplasty	69	75	22		
Macaulay (2008) [52]	Yes	Total hip arthroplasty	17	82	59	34 (29 to 42) months	Harris Hip Score
		Hemiarthroplasty	23	77	39		
Ravikumar (2000) [53] and Skinner (1989) [54]	No	Total hip arthroplasty	89	81	10	13 years	Harris Hip Score
		Hemiarthroplasty	91	82			
van den Bekerom (2010) [55]	Yes	Total hip arthroplasty	115	82	22	5 years	Harris Hip Score
		Hemiarthroplasty	137	80	16		
Total hip arthroplasty vs. osteosynthesis							
Bachrach-Lindström (2000) [24]	No	Total hip arthroplasty	50	84	20	1 year	NA
		Closed reduction and internal fixation	50	84	24		
Blomfeldt (2005a) [27], Tidermark (2003) [28], and Tidermark (2003) [29]	Yes	Total hip replacement	49	79	18	4 years	Charnley’s numerical classification
		Closed reduction and internal fixation	53	81	21		
Chammout (2012) [15]	Yes	Total hip replacement	43	78	12	17 years	Harris Hip Score
		Open reduction and internal fixation	57	79	28		
Jónsson (1996) [25]	NA	Total hip replacement	23	80^a^	22	2 years	Walking ability, pain or social function
		Closed reduction and internal fixation	24	79^a^	25		
Söreide (1979) [26]	NA	Total hip replacement	53	78	13	14.5 (12–23) months	Stinchfield’s classification system
		Reduction and internal fixation	51	78	26	14.7 (12–24) months	
Hemiarthroplasty vs. osteosynthesis							
Bjorgul (2006) [30]	NA	Hemiarthroplasty	455	82	20	6 years	NA
		Internal fixation	228	82	31		
Blomfeldt (2005b) [31]	No	Hemiarthroplasty	30	84	7	2 years	Charnley’s numerical classification
		Internal fixation	30	84	13		
Davison (2001) [10]	Yes	Thompson unipolar hemiarthroplasty	90	76^a^	21	5 years	Harris Hip Score
		Monk bipolar hemiarthroplasty	97	75^a^	26		
		Reduction and internal fixation	93	73^a^	25		
El-Abed (2005) [32]	Yes	Hemiarthroplasty	62	74	35	3 (3 to 4.5) years	Matta Scoring System
		Dynamic screw fixation	60	72	30		
Frihagen (2007) [41] and Støen (2014) [42]	No	Hemiarthroplasty	110	83	29	6 (5 to 7) years	Harris Hip Score
		Internal fixation	112	83	22		
Hedbeck (2013) [33]	No	Hemiarthroplasty	29	85	17	2 years	Charnley’s numerical classification
		Internal fixation	30	84	17		
Heetveld (2007) [34]	No	Hemiarthroplasty	109	83	17	2 years	Harris Hip Score
		Internal fixation	115	77	34		
Parker (2002) [44] and Parker (2010) [43]	No	Hemiarthroplasty	229	82	20	11 years	Charnley’s numerical classification
		Internal fixation	226	82	20		
Puolakka (2001) [35]	NA	Hemiarthroplasty	15	82	7	2 years	NA
		Internal fixation	17	81	24		
Rödén (2003) [36]	Yes	Prosthesis	47	81	28	5 years	NA
		Internal fixation	53	81	30		
Sikorski (1981) [37]	NA	Posterior Thompson	57	80	16	2 years	Pain and mobility
		Anterior Thompson	57		9		
		Internal fixation	76		21		
van Dortmont (2000) [38]	No	Hemiarthroplasty	29	84	24	16.5 (0.167 to 69.5) months	NA
		Internal fixation	31	84	3		
van Vugt (1993) [39]	Yes	Hemiarthroplasty	22	76	36	3 years	Sheperd’s pain and the hip mobility score
		Osteosynthesis	21	75	48		
Waaler Bjornelv (2012) [40]	No	Hemiarthroplasty	80	82	29	2 years	Harris Hip Score
		Internal fixation	86		22		

*NA* not available

^a^Data are shown as median numbers

As shown in Table 1, ages of participants were similar among studies and between groups of different interventions, and the majority of participants were female.

### Primary outcome measure: overall mortality

#### Total hip arthroplasty vs. hemiarthroplasty

##### Direct pairwise comparison

**Fig. 2:**
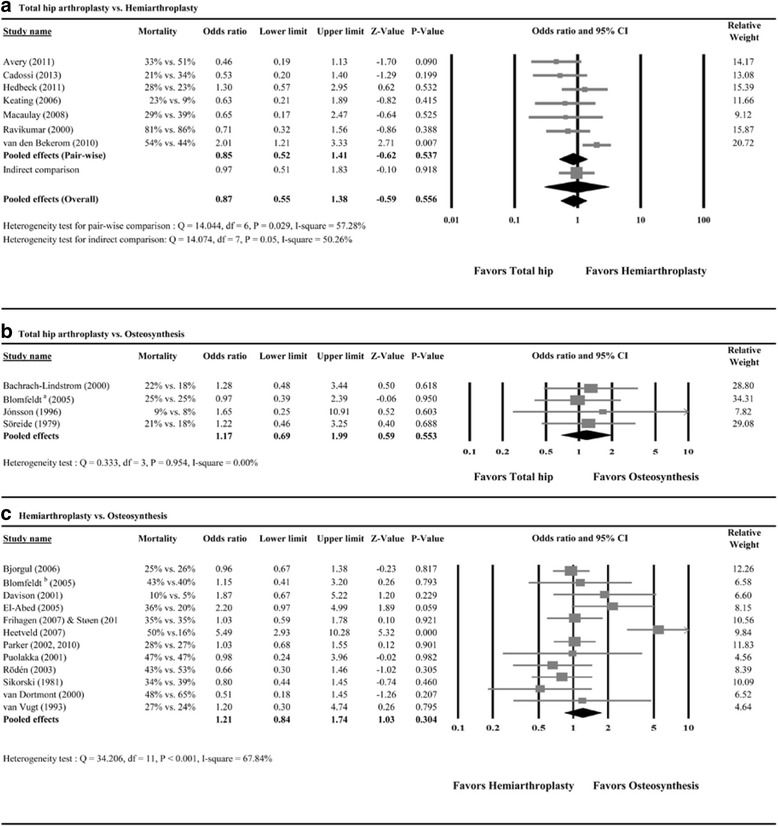
Meta-analysis forest plot for odds ratio of mortality for **a** total hip arthroplasty vs. hemiarthroplasty, **b** total hip arthroplasty vs. osteosynthesis, and **c** hemiarthroplasty vs. osteosynthesis

All seven studies reported mortality [45, 48, 49, 51–53, 55]. A random-effects model of analysis was used due to heterogeneity among the studies (*Q* = 14.044, *P* = 0.029; *I* ^2^ = 57.28%). Meta-analysis revealed no significant difference in mortality between THA and HA (pooled OR = 0.85, 95% CI 0.52 to 1.41; *P* = 0.537) (Fig. 2a).

##### Indirect comparison

Results of the adjusted indirect comparison for mortality between THA and HA are shown in Fig. 2a (OR = 0.97, 95% CI 0.51 to 1.83; *P* = 0.918). The pooled results from direct and indirect results showed no significant difference in mortality between THA and HA (pooled OR = 0.87, 95% CI 0.55 to 1.38; *P* = 0.556) (Fig. 2a). A random-effects model was used because of heterogeneity existed (*Q* = 14.074, *P* = 0.05; *I* ^2^ = 50.26%).

#### Total hip arthroplasty vs. osteosynthesis

Of the five studies, four reported patient mortality [24–27]. A fixed-effect model of analysis was used because no heterogeneity existed among the studies (*Q* = 0.333, *P* = 0.954; *I* ^2^ = 0%). The results indicated no significant difference in mortality between THA and osteosynthesis (pooled OR = 1.17, 95% CI 0.69 to 1.99; *P* = 0.553) (Fig. 2b).

#### Hemiarthroplasty vs. osteosynthesis

Of the 14 studies, 12 studies reported mortality [10, 30–32, 34–39, 41–44]. A random-effects model was used due to heterogeneity among the studies (*Q* = 34.206, *P* < 0.001; *I* ^2^ = 67.84%). There was no significant difference in mortality between HA and osteosynthesis (pooled OR = 1.21, 95% CI 0.84 to 1.74; *P* = 0.304) (Fig. 2c).

### Secondary outcome measure: revision rate

#### Total hip arthroplasty vs. hemiarthroplasty

##### Direct pairwise comparison

**Fig. 3:**
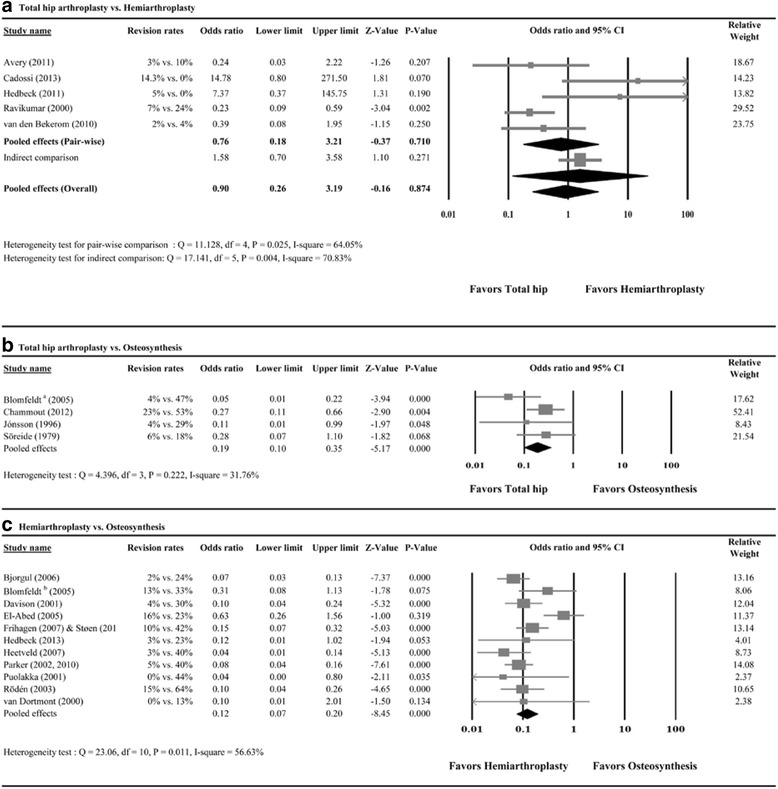
Meta-analysis forest plot for odds ratio of revision for (**a**) total hip arthroplasty vs. hemiarthroplasty, (**b**) total hip arthroplasty vs. osteosynthesis, and (**c**) hemiarthroplasty vs. osteosynthesis

Of the seven studies evaluated, five reported revision rates [45, 48, 49, 53, 55]. A random-effects model of analysis was used to evaluate heterogeneity among the studies (*Q* = 11.128, *P* = 0.025; *I* ^2^ = 64.05%). The meta-analysis showed no significant difference in revision rates between THA and HA (pooled OR = 0.76, 95% CI 0.18 to 3.21; *P* = 0.710) (Fig. 3a).

##### Indirect comparison

Results of the adjusted indirect comparison of revision rates between THA and HA are shown in Fig. 3a (OR = 1.58, 95% CI 0.70 to 3.58; *P* = 0.271). The pool of the direct and indirect results showed no significant difference in revision rates between THA and HA (pooled OR = 0.90, 95% CI 0.26 to 3.19; *P* = 0.874) (Fig. 3a). A random-effects model was used due to heterogeneity among the studies (*Q* = 17.141, *P* = 0.004; *I* ^2^ = 70.83%).

#### Total hip arthroplasty vs. osteosynthesis

Of the five studies, four reported revision rates [15, 25–27]. A fixed-effect model of analysis was used due to homogeneity among the studies (*Q* = 4.396, *P* = 0.222; *I* ^2^ = 31.76%). The pooled results showed fewer (OR = 0.19, 95% CI 0.10 to 0.35; *P* = 0.000) revisions in patients treated by THA than by osteosynthesis (Fig. 3b).

#### Hemiarthroplasty vs. osteosynthesis

Of the 14 studies, 11 studies reported revision rates [10, 30–36, 38, 41–44]. A random-effects model was used, due to heterogeneity among the studies (*Q* = 23.06, *P* = 0.011; *I* ^2^ = 56.63%). The pooled results showed fewer (OR = 0.12, 95% CI 0.07 to 0.20; *P* = 0.000) revisions for HA compared with osteosynthesis (Fig. 3c). That is to say, patients who underwent osteosynthesis were approximately eight times more likely to need a second operation.

### Sensitivity analyses and publication bias

The sensitivity tests showed no obvious influence of any individual study on the pooled estimates (Table 2). In addition, it was not possible to assess publication bias for mortality and revision rates due to the small number of studies used in the meta-analysis.

**Table 2:** Sensitivity analyses: a leave-one-out cross-validation approach

Study name	Odds ratio	Lower limit	Upper limit	*Z* value	*P* value
Mortality					
(A) Total hip arthroplasty vs. hemiarthroplasty					
Avery (2011) [45]	0.98	0.65	1.49	− 0.08	0.938
Cadossi (2013) [49]	0.95	0.61	1.46	− 0.25	0.805
Hedbeck (2011) [48]	0.83	0.52	1.33	− 0.79	0.432
Keating (2006) [51]	0.91	0.58	1.43	− 0.40	0.693
Macaulay (2008) [52]	0.90	0.58	1.41	− 0.45	0.655
Ravikumar (2000) [53]	0.91	0.57	1.45	− 0.39	0.694
van den Bekerom (2010) [55]	0.76	0.55	1.06	− 1.62	0.106
(B) Total hip arthroplasty vs. osteosynthesis					
Bachrach-Lindstrom (2000) [24]	1.13	0.60	2.11	0.39	0.700
Blomfeldt (2005) [27]	1.29	0.67	2.48	0.78	0.437
Jónsson (1996) [25]	1.14	0.66	1.98	0.47	0.641
Söreide (1979) [26]	1.15	0.62	2.16	0.45	0.655
(C) Hemiarthroplasty vs. osteosynthesis					
Bjorgul (2006) [30]	1.25	0.82	1.90	1.02	0.310
Blomfeldt (2005) [31]	1.21	0.83	1.78	0.98	0.325
Davison (2001) [10]	1.17	0.80	1.71	0.82	0.415
El-Abed (2005) [32]	1.15	0.79	1.67	0.71	0.479
Frihagen (2007) [41] and Støen (2014) [42]	1.23	0.82	1.85	1.00	0.315
Heetveld (2007) [34]	1.00	0.82	1.21	0.00	0.999
Parker (2002, 2010) [43, 44]	1.23	0.81	1.88	0.98	0.329
Puolakka (2001) [35]	1.22	0.84	1.78	1.03	0.301
Rödén (2003) [36]	1.28	0.87	1.87	1.26	0.208
Sikorski (1981) [37]	1.27	0.85	1.88	1.17	0.241
van Dortmont (2000) [38]	1.28	0.88	1.86	1.31	0.189
van Vugt (1993) [39]	1.21	0.83	1.77	0.98	0.326
Revision rates					
(A) Total hip arthroplasty vs. hemiarthroplasty					
Avery (2011) [45]	1.12	0.29	4.26	0.16	0.871
Cadossi (2013) [49]	0.63	0.20	2.00	− 0.78	0.433
Hedbeck (2011) [48]	0.69	0.20	2.38	− 0.58	0.560
Ravikumar (2000) [53]	1.29	0.38	4.39	0.41	0.684
van den Bekerom (2010) [55]	1.11	0.26	4.68	0.14	0.888
(B) Total hip arthroplasty vs. osteosynthesis					
Blomfeldt (2005) [27]	0.25	0.12	0.50	− 3.88	< 0.001
Chammout (2012) [15]	0.12	0.05	0.31	− 4.45	< 0.001
Jónsson (1996) [25]	0.20	0.10	0.38	− 4.81	< 0.001
Söreide (1979) [26]	0.17	0.08	0.34	− 4.88	< 0.001
(C) Hemiarthroplasty vs. osteosynthesis					
Bjorgul (2006) [30]	0.13	0.08	0.23	− 7.45	< 0.001
Blomfeldt (2005) [31]	0.11	0.07	0.18	− 8.49	< 0.001
Davison (2001) [10]	0.12	0.07	0.22	− 7.33	< 0.001
El-Abed (2005) [32]	0.10	0.07	0.13	− 14.80	< 0.001
Frihagen (2007) [41] and Støen (2014) [42]	0.12	0.07	0.20	− 7.48	< 0.001
Hedbeck (2013) [33]	0.12	0.07	0.20	− 8.02	< 0.001
Heetveld (2007) [34]	0.13	0.08	0.22	− 7.88	< 0.001
Parker (2002, 2010) [43, 44]	0.13	0.07	0.22	− 7.14	< 0.001
Puolakka (2001) [35]	0.12	0.07	0.21	− 8.11	< 0.001
Rödén (2003) [36]	0.12	0.07	0.21	− 7.47	< 0.001
van Dortmont (2000) [38]	0.12	0.07	0.20	− 8.11	< 0.001

### Outcome measures involving a subgroup of patients with normal cognition

**Fig. 4:**
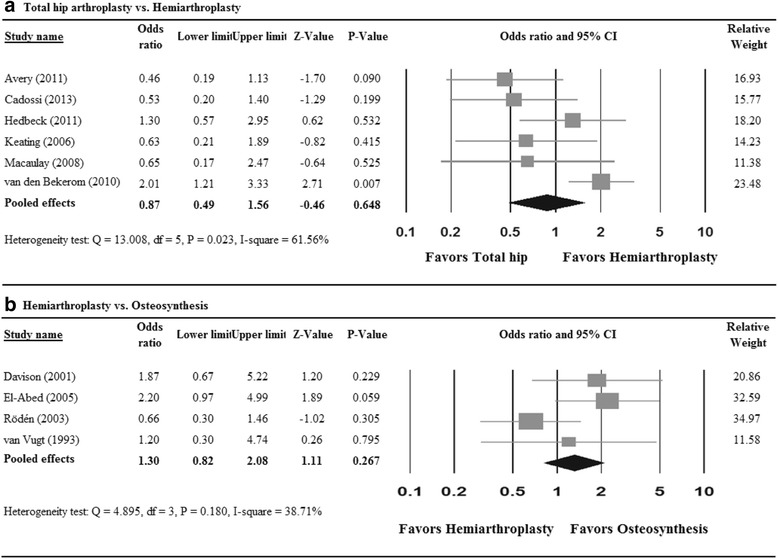
Meta-analysis forest plot of odds ratio of mortality for **a** total hip arthroplasty vs. hemiarthroplasty and **b** hemiarthroplasty vs. osteosynthesis in the subgroup of patients with no significant cognitive impairment

After excluding studies without showing normal cognition in inclusion criteria, six studies were included in the meta-analysis for the odds ratio of mortality rate for THA vs. HA. A random-effects model was used due to large heterogeneity among studies (*Q* = 13.008, *P* = 0.023; *I* ^2^ = 61.56%). The pooled odds ratio was 0.87 (95% CI 0.49 to 1.56; *P* = 0.648; Fig. 4a), suggesting that there was no significant difference in the odds ratio of mortality between patients treated with THA and HA. For the comparison between HA and osteosynthesis, four studies designed for patients with normal cognition were analyzed. A fixed-effects model was used since there was no evidence of heterogeneity among the four studies (*Q* = 4.895, *P* = 0.180; *I* ^2^ = 38.71%; Fig. 4b). No significant difference in the odds ratio of mortality was found between patients treated with HA and osteosynthesis (OR = 1.30, 95% CI 0.82 to 2.08; *P* = 0.267).

**Fig. 5:**
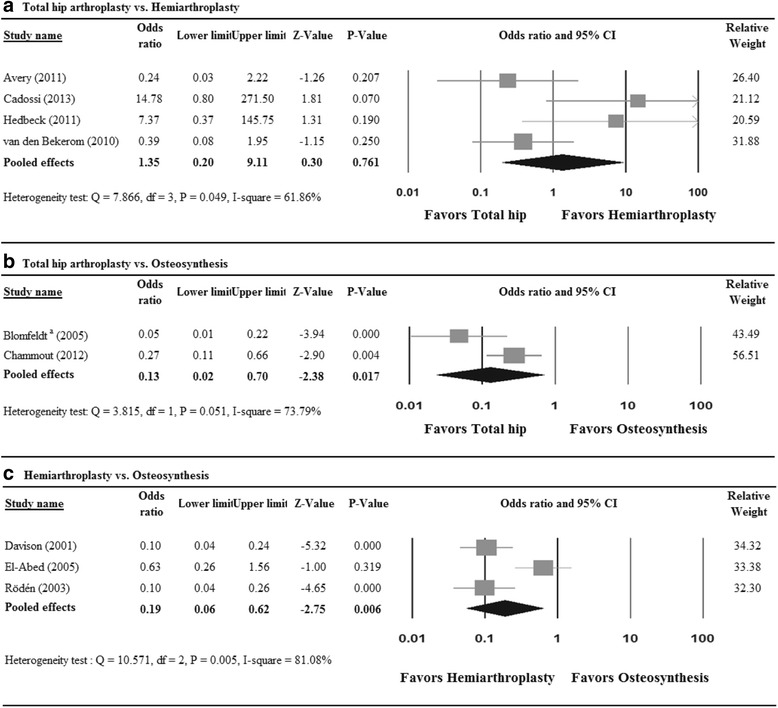
Meta-analysis forest plot of odds ratio of revision for **a** total hip arthroplasty vs. hemiarthroplasty, **b** total hip arthroplasty vs. osteosynthesis, and **c** hemiarthroplasty vs. osteosynthesis, in the subgroup of patients with no significant cognitive impairment

Four studies of patients with normal cognition were included to examine the odds ratio of revision rate for THA vs. HA. A random-effects model was used (*Q* = 7.8665, *P* = 0.049; *I* ^2^ = 61.86%). There was no difference in the odds ratio of revision between the THA and HA groups (OR = 1.35, 95% CI 0.20 to 9.11; *P* = 0.761; Fig. 5a). To compare differences in revision rate between THA and osteosynthesis groups, two studies of patients with normal cognition were included with large heterogeneity (*Q* = 3.815, *P* = 0.051; *I* ^2^ = 73.79%). Patients treated with THA had significantly lower revision rates than did those with osteosynthesis (OR = 0.13, 95% CI 0.02 to 0.70; *P* = 0.017; Fig. 5b). For the comparison between HA and osteosynthesis, three studies designed for patients with normal cognition were analyzed. A random-effects model was used due to large heterogeneity among studies (*Q* = 10.571, *P* = 0.005; *I* ^2^ = 81.08%). The pooled results showed that patients treated with HA had lower revision rates than those treated with osteosynthesis (OR = 0.19, 95% CI 0.06 to 0.62; *P* = 0.006; Fig. 5c).

### Quality assessment

**Fig. 6:**
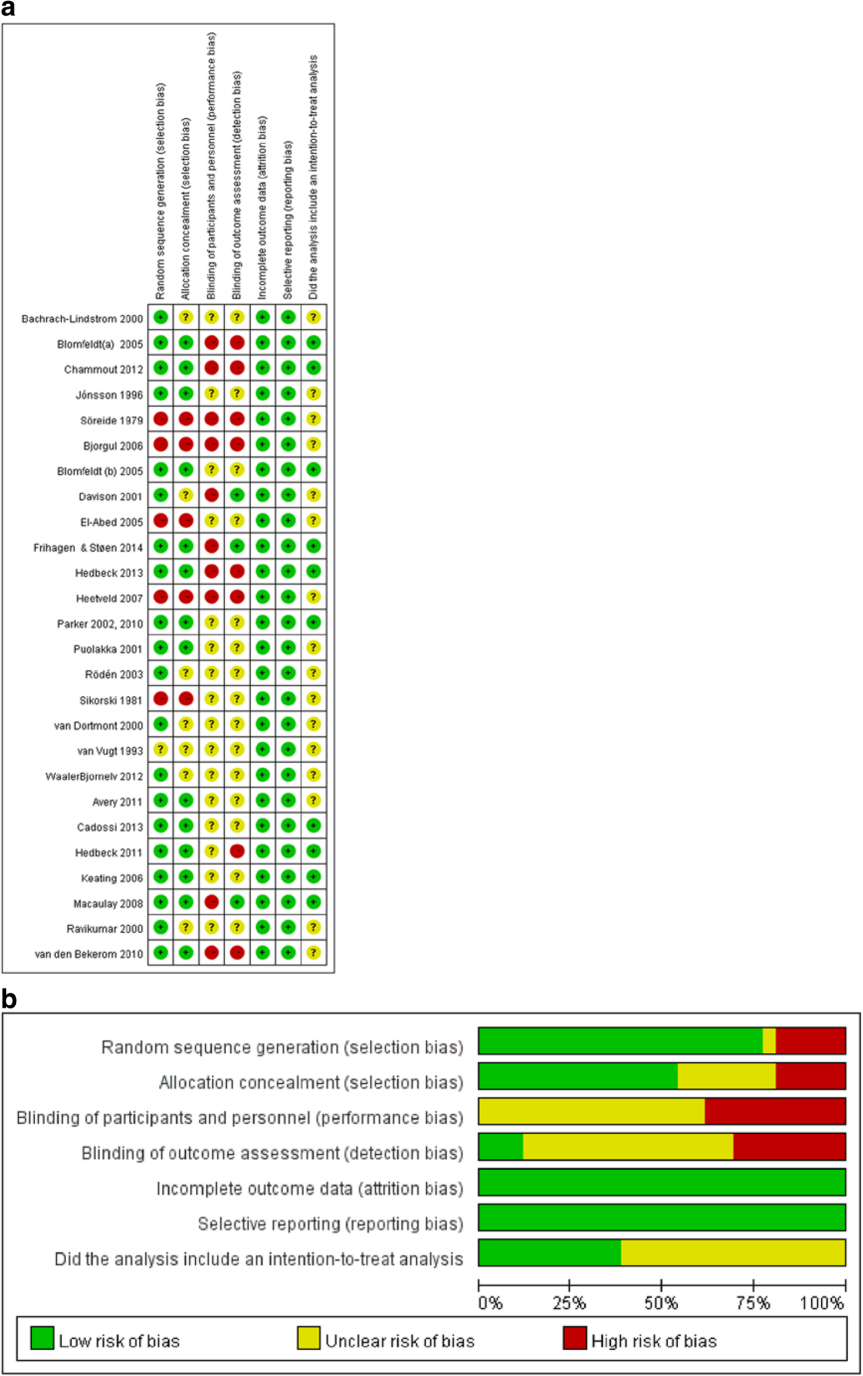
The results of quality assessment for **a** individual studies. **b** The summary of bias for all included studies

The results of quality assessment are shown in Fig. 6. In this figure, Fig. 6a shows the potential risk of bias in an individual study, and Fig. 6b shows the summary of bias for included studies. The most potential risk of bias came from performance bias and detection bias because of inadequate blinding of participants and outcome assessors.

## Discussion

This meta-analysis compared the overall mortality and revision rates between arthroplasty (HA and THA) vs. osteosynthesis for displaced femoral neck fractures in the elderly. Advanced statistical analysis (indirect comparison) was used simultaneously to compare THA and HA in order to resolve the lack of studies with head-to-head comparison between THA and HA. We also compared clinical outcomes of arthroplasty (THA and HA) vs. osteosynthesis (internal fixation) for displaced femoral neck fractures.

This meta-analysis found no significant difference in mortality rates between THA, HA, and OS. In addition, no significant difference in revision rates was found between THA and HA, but osteosynthesis had higher revision rates than either THA or HA. The additional subgroup analysis, using only studies involving elderly subjects without significant cognitive impairment, provided similar results for mortality (i.e., no difference between HA and osteosynthesis) and revision rates (no difference between THA and HA), but OS had higher revision rates than either THA or HA.

One study (two articles) showed the mean survival time of persons who died for both THA (5.3 years, range 1.3 to 9.1 years) and HA (3.8 years, range 0.003 to 7.5 years) [45, 46]. Two studies (three articles) assessed the mean survival time after intervention. Davison et al. [10] found that patients who received Thompson unipolar HA, Monk (hard-top) bipolar HA, and reduction/internal fixation had mean survival times of 61, 68, and 79 months, respectively. There was a significant difference in mean survival time between groups (*P* = 0.008). Parker et al. [43, 44] found that the patients who received HA and internal fixation had mean survival times of 2.7 years (95% CI 2.2–3.1) and 3.2 years (95% CI 2.5–3.9), respectively. No significant difference was found between groups. Due to the limitation in the number of available studies, the survival time after interventions (osteosynthesis, HA, and THA) was not included in the meta-analysis.

No meta-analysis was conducted for the functional outcome after interventions since the methods or scales for evaluating hip function were heterogeneous among the included articles (Table 1), including the Oxford Hip Score [45, 46], Harris Hip Score [10, 15, 34, 40–42, 47–49, 52–55], hip rating questionnaire [50, 51], Charnley’s numerical classification [27–29, 31, 33, 43, 44], Matta Scoring System [32], Stinchfield’s classification system [26], and Sheperd’s pain and hip mobility score [39]. In addition, no data for baseline measurement were shown in most of the studies. Therefore, it was impossible to estimate the difference in mean change before and after the intervention between the two groups.

To the best of our knowledge, this is the first meta-analysis to compare three types of interventions for displaced femoral neck fractures in one meta-analysis. Although a meta-analysis comparing THA, HA, and osteosynthesis was reported in 2012 by Gao et al. [17], the need to revisit this issue (by conducting another meta-analysis) remained because the meta-analysis by Gao et al. compared the outcomes between arthroplasty and internal fixation and thus pooled together the outcomes of HA and total hip replacement [17]. Fisher et al. [56], in their review of 3423 cases of ORIF, THA, and HA, found no differences in the 30-day mortality rates among the ORIF, HA, and THA groups, similar to our findings. ORIF and HA also resulted in a lower likelihood of developing respiratory complications than did THA [56]. A meta-analysis comparing THA and HA was reported by Burgers et al. [57]. Given the heterogeneity in surgical technique and experience over time, we felt an update of the evidence was necessary. We have updated the search, but our results were consistent with this meta-analysis that no significant difference was found in mortality and revision rates between THA and HA, but they demonstrated that THA may lead to higher dislocation rates compared with HA [57]. Therefore, it was felt that the optimal choice of arthroplasty (THA or HA) for treating femoral neck fractures had not yet been established.

It appears that the choice between arthroplasty and internal fixation in some studies was based primarily on the survival time of the implant. For example, in Davison et al. [10], HA was not recommended due to the shorter mean survival time of the implant compared with internal fixation despite the fact that internal fixation was associated with a 30% risk of failure [10]. They reported a mean patient survival which was significantly higher in the group undergoing reduction and internal fixation (79 months) compared with that with a cemented Thompson HA or a cemented Monk bipolar HA (61 and 68 months, respectively). We also found that the revision rates were lower in arthroplasty compared with internal fixation, but survival was the same among all three types of intervention (HA, THA, and osteosynthesis). These differences are likely related to the type of arthroplasty used. As in our evaluation, there was significant heterogeneity both in the implant used and the technique applied (for example, cementless HA [31, 32] vs. cemented HA [10, 30], articulation of metal on ultra-high-molecular-weight polyethylene in THR vs. metal on articular cartilage following HA [45]).

Nikitovic [6], on the basis of two systematic reviews evaluating the effectiveness of THA in comparison with HA for treatment of displaced femoral neck fractures, found a significant reduction in revision rates among patients receiving THA in comparison with HA. In addition, his recent study showed a significant improvement in functional outcome among patients receiving THA in comparison with HA, using the Harris Hip Score for the assessment. THA was favored over HA based on improvements in QoL using mobility and pain measures [6].

No meta-analysis was conducted for the QoL after interventions since the methods or scales for evaluating QoL were heterogeneous among the included articles. SF-36 was used in three studies (four articles), including three articles for THA vs. HA [45, 46, 52] and one article for HA vs. osteosynthesis [32]. EQ-5D was used in six studies (ten articles), including four articles for THA vs. HA [47, 48, 50, 51], three articles for THA vs. osteosynthesis [27–29], and three articles for HA vs. osteosynthesis [31, 33, 40]. Although we did not evaluate QoL, some studies have placed emphasis on social functioning after intervention. Jónsson et al. evaluated 50 patients with Garden stage 3 and 4 femoral neck fractures randomized for treatment using either osteosynthesis with the Hansson hook pins or THA with the Charnley prosthesis [25]. The patients were followed for up to 2 years, and their social function was evaluated using a standardized questionnaire. The authors concluded that a patient over 70 years of age who was relatively healthy, mobile, and socially independent should be considered for a primary hip prosthesis even if late complications, such as mechanical loosening, were taken into account. This conclusion was based on the fact that the majority of patients over 70 years of age are less likely to live long enough to develop implant loosening. For very old, frail, or immobile patients, however, osteosynthesis was the preferred treatment [25]. And, these findings were echoed in a more recent study from Sweden [24]. Bachrach-Lindström et al. found that a primary THA group performed better than an osteosynthesis group in weight change over time, locomotion, and pain. They also showed that primary THA could be performed safely in the elderly without increasing postoperative mortality [24].

As part of our study, we performed a sensitivity analysis and tested for homogeneity and quality. Since our analysis showed heterogeneity among the majority of studies, a random-effects model was primarily applied. We also tested for reliability based on sensitivity analysis. The direction and magnitude of the combined estimates did not change markedly with the exclusion of individual studies, indicating that our meta-analysis had good reliability. The results of quality assessment showed that the most potential risk of bias came from performance bias and detection bias because of inadequate blinding of participants and outcome assessors.

We also tested for publication bias. Although significant evidence of publication bias was found regarding differences in survival between THA and HA, we adjusted the effect of publication bias, and the adjusted point estimates of OR on mortality increased to 1.13 (95% CI 0.77 to 1.67, Fig. 2c).

In the 26 studies evaluated as part of our meta-analysis, almost all subjects had freedom of mobility or were capable of independent walking before their injury, and there was a female predominance. This is not surprising, as the incidence of proximal femoral fractures among females is two to three times greater than the incidence of such fractures in males [1]. Other risk factors for proximal femoral fractures include osteoporosis [1], a maternal history of hip fractures [58], excessive alcohol consumption and high caffeine intake [59], and physical inactivity [60], to name a few. Owing to our aging population, the risk of sustaining a proximal femoral fracture doubles every 10 years after the age of 50 [1]. Therefore, this study has clinical relevance in that it is an attempt to identify the best treatment option for these elderly patients.

Our study had several limitations. Potential performance and detection biases might exist in most of the included studies. We also did not assess functional status of the patients after the reconstructive procedures, and this was an inevitable shortcoming of this study. Notably for a patient with severe cognitive dysfunction, the lack of a surgical revision might correlate with a limited capacity for independent ambulation and with an inability to verbally express the features of a potentially symptomatic hip. We did perform an analysis on a subgroup of elderly patients without severe cognitive impairment and found no significant difference in the results regarding mortality and revision rates. But, the ways in which cognitive impairment was defined as significant or severe differed among studies. In addition, several studies only included patients with acute displaced femoral neck fractures with different time periods between fracture occurrence and admission; this ranged from 12 to 96 h. In addition, the studies included different types of femoral neck fractures, and not all studies specified the Garden stages of fractures. For all these reasons, more future studies comparing these three types of interventions are still needed to confirm our findings. Furthermore, there was significant heterogeneity among studies, especially with respect to the types of implants used for HA and THA and the types of screws used for osteosynthesis. The optimal choice of screw or reduction method (open or closed) for osteosynthesis remains unclear. Among included studies, only a few [15, 25, 45–48] chose independent living as a selection criterion, and it is arguable whether living independently or in a nursing home could have an impact on the results. Furthermore, the surgeons’ experiences and the different numbers and types of procedures performed at the various medical centers were possible confounding factors that may have affected the results and influenced the heterogeneity among studies.

## Conclusion

In conclusion, HA and THA provided similar overall mortality and revision rates, but both HA and THA had significantly lower revision rates compared with osteosynthesis. The results were not affected by excluding studies without showing normal cognition in inclusion criteria. Results of the present study provide evidence to support using hip arthroplasty for the treatment of femoral neck displaced fractures. To compare the clinical outcomes, functional outcomes, and health-related QoL between hip arthroplasty and osteosynthesis, a well-designed randomized control trial is warranted.

## Abbreviations

CI: Confidence interval
HA: Hemiarthroplasty
*I*^2^: Inconsistency index
OR: Odds ratio
QoL: Quality of life
RCT: Randomized controlled trial
THA: Total hip arthroplasty
